# Sex‐specific role of alexithymia in associations between parental bonding and mental health: A moderated mediation model

**DOI:** 10.1002/jclp.23372

**Published:** 2022-05-06

**Authors:** Ru Li, Jani Kajanoja, Linnea Karlsson, Hasse Karlsson, Max Karukivi

**Affiliations:** ^1^ Department of Psychiatry Turku University Hospital and University of Turku Turku Finland; ^2^ FinnBrain Birth Cohort Study, Department of Clinical Medicine, Turku Brain and Mind Center University of Turku Turku Finland; ^3^ Department of Psychiatry Satakunta Hospital District Pori Finland; ^4^ Center for Population Health Research, Faculty of Medicine Turku University Hospital and University of Turku Turku Finland; ^5^ Department of Adolescent Psychiatry Turku University Hospital and University of Turku Turku Finland

**Keywords:** alexithymia, anxiety symptoms, depressive symptoms, parental bonding, sex differences

## Abstract

**Objective:**

This study aimed to explore the role of alexithymia and potential sex differences in the associations between perceived parental bonding and mental health.

**Methods:**

The sample consists of 2421 parents from the FinnBrain Birth Cohort Study who completed the parental bonding instrument, the Toronto alexithymia scale, the Edinburgh postnatal depression scale, and the anxiety subscale of the symptom checklist‐90. Moderated mediation analyses were conducted to examine the possible mediating role of alexithymia and moderating role of sex in the associations between parental bonding and depressive/anxiety symptoms.

**Results:**

Alexithymia was found to be a potential mediator and sex be a moderator in the relations between perceived dysfunctional parental bonding and the psychological symptoms. Specifically, dysfunctional paternal bonding, especially paternal overprotection, had stronger indirect effects (via alexithymia) on the psychological symptoms in males.

**Conclusions:**

This study indicates the importance of alexithymia in the parenting‐related mental health impacts and highlights the significance of paternal bonding for the development of alexithymia and mental health problems in male populations. The findings improve the limited understanding of sex‐related parental factors for alexithymia and mental health problems. Future studies in longitudinal designs are warranted to clarify the causal process of the mediation.

## 1 INTRODUCTION

Parental bonding has long been considered to play a crucial role in mental illnesses including depression and anxiety in adults (Duggan et al., 1998; Heider et al., 2006; Kullberg et al., 2020). It is suggested that strategies and a capacity to regulate emotions can be facilitated during childhood via sufficiently good‐quality interaction with parents (Brumariu, 2015; de Cock et al., 2017). Parental bonding refers to relationship quality or attachment style perceived by parents during one's childhood (Parker et al., 1979). Functional or adequate parental bonding reflects secure relationships with an intimate, warm, and supportive relationship without excessive or intrusive control. According to the theory of attachment by Bowlby, the absence of secure attachment renders one vulnerable to later mental health problems (Bretherton, 1992; Mikulincer & Shaver, 2012).

Alexithymia, a personality construct characterized by difficulties in identifying (DIF) and describing feelings (DDF), externally oriented thinking (EOT) style, and a scarcity of imagination (Sifneos, 1973), may be a crucial factor in the mental health influences of perceived parental bonding. Dysfunctional parental bonding (DysPB) experiences (i.e., perceived inadequate parental care [PC] and/or undue parental overprotection [PO]) during childhood have been reported to be associated with alexithymia (De Panfilis et al., 2003; Fukunishi et al., 1999; Kooiman et al., 2004; Mannarini et al., 2018; Nemiah, 1977; Pedrosa Gil et al., 2008). Additionally, although not classified as a psychiatric disorder, alexithymia has been related to a variety of mental illnesses including depression and anxiety (Dorard et al., 2017; Honkalampi et al., 2000; Marchesi et al., 2005), which implies that alexithymia may be a factor in mediating the well‐established links between DysPB and the development of psychological distress. Existing research has indicated a mediating role of alexithymia in associations of negative experiences in early life such as child sexual abuse and emotional neglect with adult mental health problems including depressive and anxiety symptoms (Brown et al., 2016; Hébert et al., 2018). A study by Lyvers et al. (2019) suggested that alexithymia mediated the associations between parental bonding and problematic alcohol use. However, evidence as to the role of alexithymia in mediating the relations between DysPB experiences and psychological symptoms is scarce.

Moreover, interestingly, previous research showed differential patterns of the association between parental bonding and alexithymia. For example, some studies with normal populations indicated a weaker impact of paternal bonding on alexithymia compared to maternal bonding (Fukunishi, 1998; Fukunishi et al., 1997, 1999; Karukivi et al., 2011), but more significant associations between paternal bonding and alexithymia have been found in research with clinical samples (Kooiman et al., 1998, 2004), which implied that their relations may be influenced by individual characteristics.

Perceived parental bonding in childhood may have differential effects on later alexithymia and mental health problems between sex. First, there are sex differences in the prevalence of alexithymia, with generally more alexithymic features among males (Franz et al., 2008; Kokkonen et al., 2001; Mattila et al., 2006; Salminen et al., 1999). Second, sex differences in learning emotional expressions from parents during one's early life have been observed (Chaplin & Aldao, 2013). Findings from previous studies on the associations of parental bonding with alexithymia differ between males and females. For instance, a study using pair‐wise analyses showed that the relations between maternal bonding and alexithymia in male adolescents were almost as strong as in females, but paternal bonding seemed to have stronger relations to alexithymia in females (Karukivi et al., 2011). In addition, there is strong evidence that females tend to be more susceptible to psychological distress including the onset of depression and anxiety than males (McHenry et al., 2014; Salk et al., 2017). However, there are previous studies suggesting no sex differences in the associations between parenting factors (e.g., monitoring and parental control) and adolescent depression and anxiety (Hamza & Willoughby, 2011; Yap et al., 2014).

Understanding the sex‐specific role of an alexithymic trait in mental health problems related to experienced parental bonding during childhood would be relevant to identifying vulnerable individuals. To our knowledge, no studies have determined the sex‐moderated indirect effects of parental bonding on mental health problems via alexithymia, which makes it an area worthy of exploration. Thereby, the aim of this study is to investigate the role of alexithymia in the mental health impacts of DysPB experiences during childhood, as well as the possible sex differences in their associations. We hypothesized that both dysfunctional maternal bonding (DysMB) and dysfunctional paternal bonding (DysPB) have direct and indirect relations to mental health (depressive and anxiety) symptoms, and such indirect relations would be mediated by alexithymia. Furthermore, sex was expected to moderate the direct and indirect (alexithymia‐mediated) mental health effects of DysPB.

## 2 MATERIALS AND METHODS

### 2.1 Study participants

The present study is a cross‐sectional sub‐study based on the FinnBrain Birth Cohort Study (*N* = 3808 families), a prospective cohort study exploring the impacts of prenatal and early life stress on child brain development and health (www.finnbrain.fi, also see Karlsson et al., 2018, for more detailed information). Participants were recruited in Finland between December 2011 and April 2015 from maternal welfare clinics in the South‐Western Hospital District and the Åland Islands. The Ethics Committee of the Hospital District of Southwest Finland has approved the study protocol (June 14, 2011 ETMK:57/180/2011 § 168).

Data on parental bonding was collected at Gestational Week 24. Data on alexithymia, depressive, and anxiety symptoms were collected 6 months after delivery. Overall, 1652 mothers and 852 fathers from the FinnBrain Birth Cohort Study had completed the questionnaires on parental bonding, alexithymia, and psychological symptoms. Of these, 83 participants with missing demographic data were excluded, and thus a sample consisting of 2421 participants (1599 females and 822 males) was used in this study.

### 2.2 Measures

#### 2.2.1 Demographics

For this study, the demographic information about the participants included age, sex, and education divided into three classes (low: high school or lower; mid: vocational/upper secondary school degree; and high: applied sciences/university degree), which is collected at Gestational Week 14.

#### 2.2.2 Parental bonding

Parental bonding was measured with the parental bonding instrument (PBI) (Parker et al., 1979), which is used to retrospectively assess parental attitudes and behaviors toward the respondents during their first 16 years of life. It is a self‐report questionnaire consisting of 25 items rated on a 4‐point Likert scale from 0 to 3 and has shown satisfactory reliability and validity (Parker, 1989). The PBI comprises two subscales (12 items for experienced care and 13 items for overprotection) applied to mothers and fathers, which form maternal care and overprotection as well as PC and PO. There are bipolar dimensions for each subscale. The care subscale assesses emotional warmth, support, and closeness at a positive pole, versus emotional neglect, indifference, and coldness at a negative pole, while the positive pole for overprotection is characterized by the promotion of independence and autonomy, and the negative pole by excessive contact, overcontrol, and intrusion.

Following the principle of parsimony, the scores of the items at the positive pole for both subscales were reversed thus yielding two variables representing DysPB (higher values indicating lower PC and/or higher PO), which were used in this study. The Cronbach's *α* was 0.91 for DysMB and 0.92 for DysPB.

#### 2.2.3 Alexithymia

The Toronto alexithymia scale (TAS‐20) is one of the most prevalent validated self‐report scales used to measure alexithymic features (Bagby et al., 1994; Joukamaa et al., 2001; Taylor et al., 2003). It consists of 20 items divided into three subscales: DIF, DDF, and EOT. The items are rated with a 5‐point Likert scale (1 = strongly disagree, 5 = strongly agree) and the total score lies between 20 and 100. In this study, the validated Finnish TAS‐20 used in this study (Joukamaa et al., 2001) showed good internal consistency with a global Cronbach's *α* = 0.83 for the TAS‐20 total scores (*α* = 0.81 for the DIF subscale, *α* = 0.77 for DDF, and *α* = 0.65 for EOT).

#### 2.2.4 Depressive and anxiety symptoms

Depressive symptoms were assessed using the Edinburgh postnatal depression scale (EPDS), a widely used 10‐item self‐report questionnaire with high validity and sensitivity employed for screening postpartum depressive symptoms (Cox et al., 1987; Gibson et al., 2009). It is also a reasonably valid tool to be administered to fathers (Edmondson et al., 2010). Moods and other depressive symptoms in the previous week are rated. Each question is scored from 0 to 3 and the total score ranges from 0 to 30 points. The Finnish version of the EPDS has been validated and used for decades (Tamminen, 1990), and it showed good internal consistency in this study with the Cronbach's *α* = 0.84.

Anxiety symptoms were measured using the symptom checklist‐90 (SCL‐90), a developed self‐report questionnaire to assess the intensity of symptoms on multidimensional subscales (Derogatis et al., 1973). In this study, the anxiety subscale of the validated Finnish SCL‐90 (Holi et al., 1998) was used for assessing the intensity of anxiety experienced in the previous month, which consists of 10 items rated on a 5‐point scale from 0 to 4 (Cronbach's *α* = 0.85).

### 2.3 Statistics

Statistical analyses were conducted using IBM SPSS 25.0. Normality of distribution within variables was examined visually and by the Shapiro–Wilk test. Descriptive statistics were calculated for the study variables stratified by sex. Categorical variables were compared with the *χ* ^2^ test and continuous variables with the Mann–Whitney *U* test due to non‐normally distributed data. For quantifying the strength of the relations between the study variables, Spearman's correlation coefficient (*ρ*) was used.

**Figure 1:**
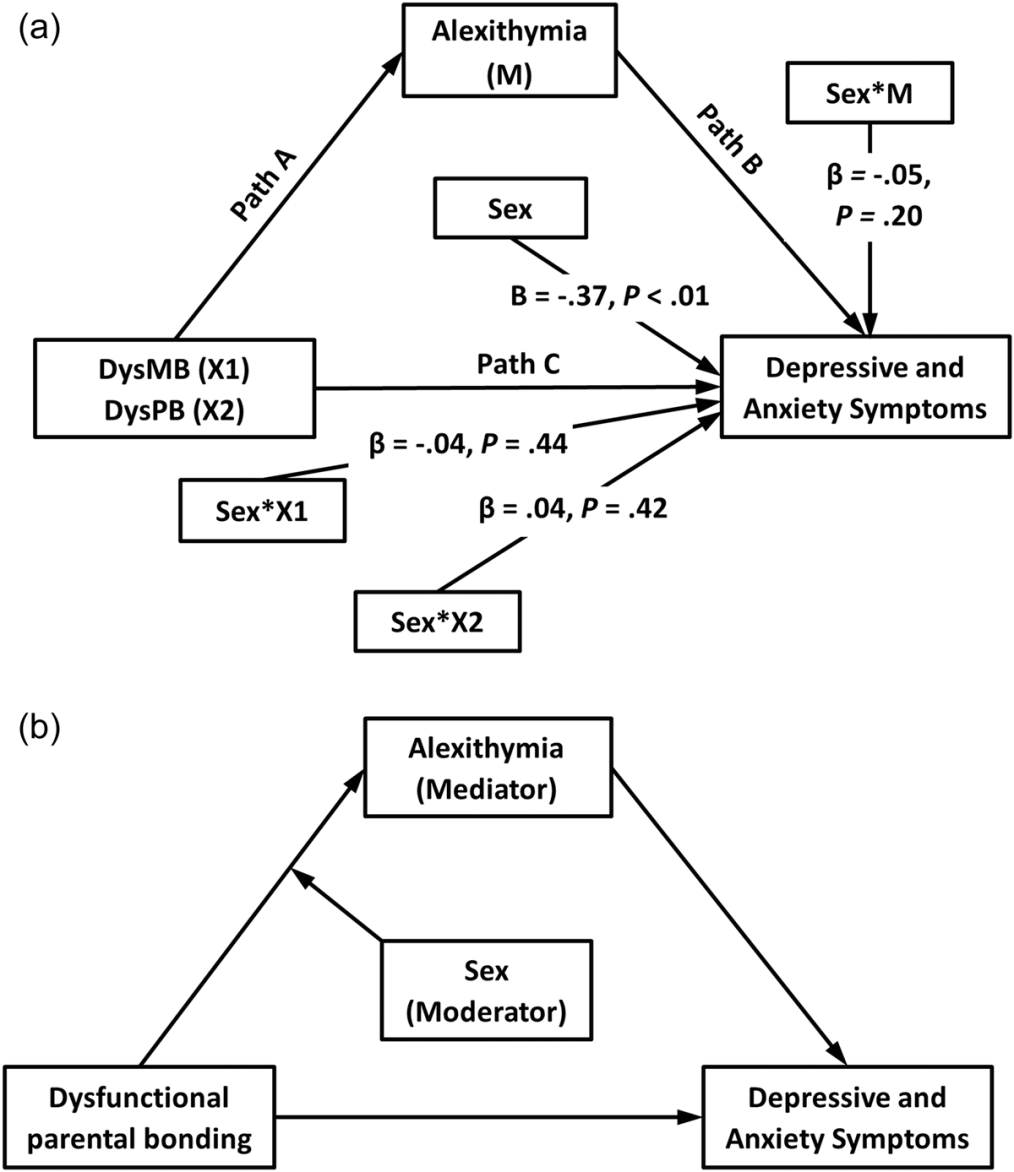
Preliminary tests for the moderation effects of sex in the conceptual mediation model (a) and the research moderated mediation model (b). DysMB, dysfunctional maternal bonding; DysPB, dysfunctional paternal bonding.

First, the mediation effects of alexithymia on the relations between DysPB (both DysMB and DysPB) and the depressive and anxiety symptoms were tested by using Model 4 of the PROCESS 3.5 macro for SPSS (Hayes, 2017), controlling for age, sex, and education levels. Second, the moderation effects of sex on the mediation model were preliminarily tested by conducting the moderated mediation (or conditional process) analyses with Model 59 of the PROCESS macro. Although sex had significant relations to the psychological symptoms, no significant associations were observed between the interaction terms (sex × DysMB/DysPB/alexithymia) and the symptoms, indicating that sex did not moderate Path B (alexithymia → psychological symptoms) nor path C (DysPB → psychological symptoms) of the conceptual model (as depicted in Figure 1a). Thus, Model 7 of the macro was used (as depicted in Figure 1b). Simple slope analysis was used to test the moderating role of sex on the single path. The mediation effects and the moderated mediation effects were determined by applying the bootstrapping method with 10,000 samples adopted to compute 95% bootstrap confidence intervals (CIs). The bootstrapped 95% CI that does not include zero indicates a significant effect (Hayes & Rockwood, 2019).

To be specific, if there is a significant moderated mediation effect for DysMB or DysPB, we further exploratively tested whether the corresponding subscales of the PBI (i.e., maternal care/PC and PO) play a role in the sex‐specific associations.

## 3 RESULTS

### 3.1 Descriptive statistics and correlations

Table 1 presents the characteristics of the participants and Spearman's correlation matrix for the study variables. Age and the TAS‐20 score (alexithymia) were presented as mean and standard deviation. The scores for DysMB and DysPB, the EPDS, and SCL‐90 scores (depressive and anxiety symptoms) were presented as the median and interquartile range due to non‐normal distributions. The *χ* ^2^ test showed that females were more highly educated compared to males. The Mann–Whitney *U* test showed significantly higher alexithymia, but lower DysMB/DysPB and depressive symptoms in males, compared with females. According to Spearman's correlation matrix, age, depressive and anxiety symptoms, and alexithymia were positively correlated with perceived DysPB.

**Table 1:** Descriptive statistics, sex differences in all variables, and Spearman's correlation for continuous variables.^a^

	Females (*N* = 1599)	Males (*N* = 822)	Total (*N* = 2421)	1	2	3	4	5	6
Education									
Low	351 (31.5%)	504 (42.7%)	855 (35.3%)*						
Mid	473 (29.6%)	237 (28.8%)	710 (29.3%)						
High	622 (38.9%)	234 (28.5%)	856 (35.4%)*						
Mean (SD)									
1. Age	30.7 (4.3)	32.4 (5.4)	31.3 (4.8)**	–	−0.04	0.07**	0.06**	−0.03	−0.03
2. TAS‐20	39.8 (9.2)	42.9 (9.6)	40.8 (9.5)**		–	0.23**	0.23**	0.36**	0.30**
Median (IQR)									
3. DysMB	16.2 (11.0)	14.4 (8.9)	15.6 (10.3)*			–	0.59**	0.26**	0.22**
4. DysPB	18.2 (11.5)	16.8 (10.7)	17.8 (11.2)*				–	0.25**	0.21**
5. EPDS	4.0 (6.0)	2.0 (4.0)	3.0 (5.0)**					–	0.61**
6. SCL‐90	1.0 (4.0)	1.0 (4.0)	1.0 (4.0)						–

*Note*: *χ^2^* Test for comparisons of the categorical variables between men and women. Mann–Whitney *U* test for comparisons of the continuous variables between men and women.

Abbreviations: DysMB, dysfunctional maternal bonding; DysPB, dysfunctional paternal bonding; EPDS, Edinburgh postnatal depression scale; IQR, interquartile range; SCL‐90, symptom checklist‐90; TAS‐20, Toronto alexithymia scale.

^a^ Education: Low = high school or lower; mid = vocational/upper secondary school degree; and high = applied sciences or university degree.

* *p* < 0.05.

** *p* < 0.01.

### 3.2 Mediation analyses

The results of mediation effects are presented in the Model 1 (mediation‐only) in Table 2. For the first path, both DysMB (*β* = 0.142, *p* < 0.001) and DysPB (*β* = 0.144, *p* < 0.001) were positively associated with alexithymia after controlling for age, sex, and education. For the second path, as shown in Table 2 (the lower part), alexithymia was positively related to the depressive (*β* =0.382, *p* < 0.001) and anxiety symptoms (*β* = 0.318, *p* < 0.001). Moreover, the bootstrapped 95% CI for the indirect of effects of DysPB on the depressive (*β* = 0.054, SE = 0.011, 95% CI = [0.034, 0.075] for DysMB; *β* = 0.055, SE = 0.010, 95% CI = [0.036, 0.075] for DysPB) and anxiety (*β* = 0.045, SE = 0.009, 95% CI = [0.029, 0.062] for DysMB; *β* = 0.046, SE = 0.009, 95% CI = [0.029, 0.064] for DysPB) symptoms did not include zero, which suggested a significant mediating effect of alexithymia on these associations. The model explained 23% of variance in the depressive symptoms (*R* ^2^ = 0.23, *F* [6, 2414] = 117.36, *p* < 0.001), and 15% in the anxiety symptoms (*R* ^2^ = 0.15, *F* [6, 2414] = 70.29, *p* < 0.001).

**Table 2:** Mediation models and moderated mediation models.^a^

	*β*	SE	Bootstrapped 95% CI		*p*	*β*	SE	Bootstrapped 95% CI		*p*
			LLCI	ULCI				LLCI	ULCI	
Outcome: Alexithymia (M)	Model 1 (mediation‐only)					Model 2 (moderated mediation)				
Constant	−0.102	0.082	−0.266	0.063	0.213	−0.113	0.082	−0.278	0.054	0.167
Age	−0.049	0.020	−0.088	−0.010	0.015	−0.055	0.020	−0.094	−0.015	0.007
Education	−0.180	0.024	−0.227	−0.132	<0.001	−0.179	0.024	−0.227	−0.132	<0.001
Sex	0.345	0.042	0.261	0.426	<0.001	0.356	0.042	0.271	0.439	<0.001
DysMB (X1)	**0.142**	**0.022**	**0.093**	**0.192**	**<0.001**	0.123	0.069	−0.035	0.274	0.076
DysPB (X2)	**0.144**	**0.022**	**0.097**	**0.192**	**<0.001**	−0.086	0.068	−0.229	0.061	0.207
Interaction (X1 × sex)						**0.015**	**0.053**	**−0.097**	**0.139**	**0.774**
Interaction (X2 × sex)						**0.176**	**0.050**	**0.065**	**0.282**	**<0.001**
Outcome	Depressive symptoms (Y1)					Anxiety symptoms (Y2)				
Constant	0.477	0.077	0.318	0.635	<0.001	0.110	0.081	−0.046	0.273	0.172
Age	0.003	0.019	−0.033	0.041	0.865	−0.033	0.020	−0.071	0.005	0.094
Education	0.015	0.023	−0.029	0.060	0.505	0.026	0.024	−0.020	0.072	0.269
Sex	−0.378	0.040	−0.451	−0.302	<0.001	−0.121	0.042	−0.202	−0.043	0.004
DysMB (X1)	0.104	0.021	0.053	0.154	<0.001	0.094	0.022	0.045	0.143	<0.001
DysPB (X2)	0.102	0.021	0.055	0.150	<0.001	0.087	0.022	0.039	0.135	<0.001
Alexithymia (M)	**0.382**	**0.019**	**0.339**	**0.426**	**<0.001**	**0.318**	**0.020**	**0.269**	**0.367**	**<0.001**

*Note*: *N* = 2421; bootstrapped sample size = 10,000. Sex: 1 = females; 2 = males.

The focal factors for the mediation and moderated mediation are highlighted in bold.

Abbreviations: CI, confidence interval; DysMB, dysfunctional maternal bonding; DysPB, dysfunctional paternal bonding; LLCI, lower limit confidence interval; ULCI, upper limit confidence interval.

^a^ Mediation models: Model 1 → Y1/Y2. Moderated mediation models: Model 2 → Y1/Y2.

### 3.3 Moderated mediation analyses

Table 2 (Model 2) demonstrates the results of moderated mediation analyses, controlling for age, sex, and education. There was no significant interaction between sex and DysMB in the path to alexithymia (*p* = 0.774). However, significant interaction effects of sex and DysPB on alexithymia were found (*β* = 0.176, *p* < 0.001), which indicated that the link between DysPB and alexithymia was moderated by sex. The model explained 12% of variance in alexithymia (*R* ^2^ = 0.12, *F* [7, 2413] = 48.63, *p* <0.001).

**Figure 2:**
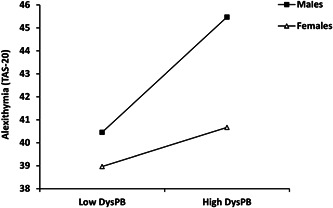
Conditional effects of dysfunctional paternal bonding (DysPB) on alexithymia depending on sex. Low = mean − 1SD; high = mean + 1SD. TAS‐20, Toronto alexithymia scale.

The simple slopes analysis showed a moderating role of sex in the single path from DysPB to alexithymia. Alexithymia was higher in males compared to females at both low DysPB (*p* = 0.039) and high DysPB (*p* < 0.001). Moreover, a steeper slope for males suggested that perceived DysPB during childhood had a stronger effect on alexithymia among males (*β* = 0.266 vs. *β* = 0.090 for females, *p* < 0.001) (Figure 2).

Additionally, as presented in Table 3, the indirect effect of DysPB on the psychological symptoms was found to be significantly stronger in males than females (pairwise contrast = 0.067 for depressive symptoms and 0.056 for anxiety symptoms), with the bootstrapped 95% CI excluding zero. Still, the mediation effect of alexithymia between DysMB and the depressive and anxiety symptoms was not conditional on sex, with both bootstrapped 95% CI including zero.

**Table 3:** Indirect effects of dysfunctional parental bonding via alexithymia on the psychological symptoms in females and males, and explorative moderated mediation analyses for paternal bonding subscales.^a^

Path	Sex	Depressive symptoms (Y1)				Anxiety symptoms (Y2)			
		Effect	SE	Bootstrapped 95% CI		Effect	SE	Bootstrapped 95% CI	
				LLCI	ULCI			LLCI	ULCI
DysMB → alexithymia → Y	Females	0.053	0.012	0.031	0.076	0.044	0.010	0.026	0.064
	Males	0.059	0.021	0.020	0.100	0.049	0.018	0.016	0.086
	Contrasts	0.006	0.023	−0.038	0.052	0.005	0.019	−0.031	0.044
DysPB → alexithymia → Y	Females	0.034	0.011	0.014	0.057	0.029	0.009	0.011	0.048
	Males	0.102	0.019	0.064	0.140	0.085	0.017	0.053	0.118
	Contrasts	**0.067**	**0.022**	**0.025**	**0.110**	**0.056**	**0.018**	**0.021**	**0.092**
PC → alexithymia → Y	Females	−0.058	0.011	−0.080	−0.037	−0.049	0.009	−0.069	−0.031
	Males	−0.094	0.017	−0.128	−0.063	−0.078	0.015	−0.108	−0.051
	Contrasts	−0.036	0.019	−0.075	0.001	−0.030	0.016	−0.061	0.002
PO → alexithymia → Y	Females	0.015	0.011	−0.006	0.036	0.013	0.009	−0.005	0.030
	Males	0.068	0.017	0.035	0.103	0.057	0.015	0.029	0.087
	Contrasts	**0.053**	**0.020**	**0.014**	**0.093**	**0.044**	**0.017**	**0.012**	**0.079**

*Note*: Significant differences in effects between females and males are highlighted in bold.

Abbreviations: CI, confidence interval; DysMB, dysfunctional maternal bonding; DysPB, dysfunctional paternal bonding; LLCI, lower limit confidence interval; PC, paternal care; PO, paternal overprotection; ULCI, upper limit confidence interval.

^a^ *N* = 2421; bootstrapped sample size = 10,000.

**Figure 3:**
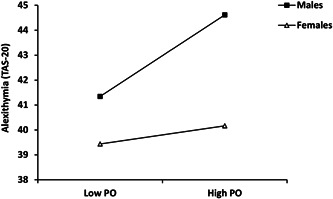
Conditional effects of paternal overprotection (PO) on alexithymia depending on sex. Low = mean − 1SD; high = mean + 1SD. TAS‐20, Toronto alexithymia scale.

Based on the results, we further exploratively tested whether both subscales of the PBI, that is PC and PO, play a role in this sex‐moderated mediation model. In the first path of the mediation model, both PC (*p* = 0.365) and PO (*p* = 0.127) were not significantly related to alexithymia in the whole sample controlling for sex; however, the analysis of the single‐slope indicated a moderating role of sex in this path. A significant effect of PO on alexithymia in males (*β* = 0.173, 95% CI = [0.095, 0.252], *p* < 0.001), but not in females (*β* = 0.038, 95% CI = [−0.010, 0.086], *p* =0.118) (as illustrated in Figure 3). The effect of PC on alexithymia was not moderated by sex.

Table 3 presents the corresponding results for the conditional indirect effects of PC and PO on the depressive and anxiety symptoms. The indirect effect of PO, but not PC, on the psychological symptoms, was conditionally depending on sex, with the bootstrapped 95% CI excluding zero indicating a significantly stronger effect in males compared to females (pairwise contrast = 0.053 for depressive symptoms and 0.044 for anxiety symptoms).

## 4 DISCUSSION

The present study investigated the associations between parental bonding experienced during childhood, alexithymia, and mental health, as well as sex differences in such associations. By conducting a moderated mediation model, alexithymia showed a mediating effect on the indirect relations between perceived DysPB and depressive and anxiety symptoms. Furthermore, sex moderated the link between alexithymia and DysPB, but not DysMB, in turn, moderating the indirect effects of DysPB via alexithymia on the depressive and anxiety symptoms. These results suggested a sex‐specific role of alexithymia in the associations between DysPB and mental health.

### 4.1 Relations between parental bonding and mental health, and alexithymia as a mediator

Consistent with previous evidence that perceived parental bonding during childhood is associated with mental health (Kullberg et al., 2020; van der Bruggen et al., 2008), our study showed a significant direct link between DysPB experiences and depressive and anxiety symptoms. Specifically, both DysMB and DysPB were found to be related to psychological symptoms with largely similar effects. Although most existing studies highlight the importance of maternal bonding or mother–child attachment (Abbaspour et al., 2021; Eun et al., 2018), perceived dysfunctional parenting styles from fathers are also significant risk factors for mental illnesses (Eun et al., 2018; Hamza & Willoughby, 2011). For example, research with large population samples has demonstrated that parenting received from mothers and fathers during childhood was equally associated with adult mental health outcomes (Kendler et al., 2000; Otowa et al., 2013).

In addition to the direct link between parental bonding and mental health, there were also indirect effects of perceived DysPB on mental health problems with alexithymia as a potential mediator. In other words, DysPB experienced during childhood may contribute to the development of alexithymic features, in turn, exacerbating mental health problems. For the first path, in accordance with most previous studies that maternal bonding has been linked to alexithymia in most previous studies (De Panfilis et al., 2003; Fukunishi et al., 1997, 1999; Mason et al., 2005), the present study indicated significant relations between DysMB and alexithymia. Additionally, several studies have reported relations between fathers' parenting styles from fathers and alexithymia, supporting our findings that DysPB was also associated with alexithymia (Kooiman et al., 2004; Pedrosa Gil et al., 2008). However, maternal bonding seems to have a stronger impact on alexithymia in nonclinical samples (Fukunishi et al., 1997, 1999; Karukivi et al., 2011), which is in contrast to the current finding that DysMB and DysPB have largely equal relations to alexithymia when controlling for sex. This may be due to our sample selection, that is, a relatively large sample from a population‐based birth cohort. For the second path, the significant link of alexithymia to the depressive and anxiety symptoms observed in this study is in line with the reviewed research indicating significant relations between alexithymia and various mental disorders (Honkalampi et al., 2000; Marchesi et al., 2005).

Our finding suggests a plausible explanatory mechanism underlying the mental impacts of parental bonding. It is indicated that an insecure parent–child attachment in childhood is associated with decreased emotion identification and regulation, which may manifest as an alexithymic feature in adulthood (Brumariu et al., 2012; Colle & Del Giudice, 2011; Steele et al., 2008). On one hand, deficits in emotion regulation tend to be associated with an increase in negative affect (Berking & Wupperman, 2012). On the other hand, alexithymic individuals have difficulties in linking affective states to specific memories or situations, thus leading to more negative emotions due to a lack of adequate coping strategies, which may further bring about mental health problems (Rieffe et al., 2010; Taylor et al., 1997).

### 4.2 Sex as a moderator showing sex differences

In line with the evidence of sex differences in the presentation of psychological distress (McHenry et al., 2014; Salk et al., 2017), our study demonstrated that sex was significantly related to both depressive and anxiety symptoms. However, in the preliminary analysis, no moderating role of sex was observed in the direct links between DysPB (both DysMB and DysPB) and the depressive and anxiety symptoms, indicating no sex differences in the direct mental effects of parental bonding. There are previous studies suggesting that the relations between parenting factors and depression/anxiety were similar for males and females (Hamza & Willoughby, 2011; McLeod et al., 2007; Yap, et al., 2014). Nevertheless, the indirect effects of perceived DysPB on the psychological symptoms showed significant sex differences.

Sex was found to moderate the indirect relations between DysPB and the psychological symptom in the current study. More specifically, the relations between DysPB and alexithymia were moderated by sex, which, in turn, moderated the indirect effects of DysPB on the depressive and anxiety symptoms. Such conditional effects were not observed in the relation between DysMB and alexithymia. Surprisingly, although significant sex differences were found in both depressive and anxiety symptoms, with more psychological symptoms in females, DysPB was indicated to have stronger indirect effects on psychological symptoms in males. Therefore, the sex‐moderated mediation effects of alexithymia may explain the sex differences shown in some previous studies by applying the same PBI measure. For example, perceived paternal bonding was indicated as a better parental predictor than maternal bonding for the increases in depression in males (Howard, 1981; Shibata et al., 2016). In a study by Enns et al. (2002), males with psychiatric disorders, such as depression, dysthymia, general anxiety disorder, or panic disorder reported more DysPB than DysMB, especially a lack of PC. Although research on the role of father–child attachment is scarce, there are possible theoretical explanations for the relatively strong mental effects of paternal bonding towards males. It is suggested that father–child attachment or interactions are characterized by greater emotional arousal, a fundamental aspect of emotion perception and regulation (Brand & Klimes‐Dougan, 2010; Brumariu, 2015; Deckert et al., 2020), which shares commonalities with alexithymia referring to DIF and expressing emotions and thus, supporting the relationship between paternal bonding and alexithymia. In addition, there is evidence suggesting that males with early insecure attachment tend to adopt avoidant coping strategies (Brumariu, 2015; Del Giudice, 2009), and alexithymia, to a certain extent, reflects an avoidance‐oriented coping style (J. D. Parker et al., 1998). This may account for the stronger mediation effect of alexithymia in males. Future studies would benefit from exploring potential predictors and mediators for explaining higher levels and prevalence of emotional distress in female populations.

Furthermore, when considering the subscales for paternal bonding of the PBI, PO appeared to be the dominant factor contributing to the effects of paternal bonding in our model. Similar to several previous studies, our results for the sample as a whole demonstrated that PO was not associated with alexithymia (De Panfilis et al., 2003; Fukunishi et al., 1997, 1999). Nonetheless, the associations between PO on alexithymia and the psychological symptoms vary by sex. In males, perceived PO during childhood was significantly linked to alexithymia, which, in turn, had relations to the depressive and anxiety symptoms. This is partially supported by some of the previous findings. For instance, one study has suggested that PO was related to alexithymia in males (Karukivi et al., 2011). In addition, males with a higher level of mental health problems have reported more perceptions of PO (Shibata et al., 2016). PO refers to harsh and hostile discipline, deterring children from expressing feelings and ideas as well as hindering the capacity for self‐regulation, which may account for the effect of PO perceived during childhood on later alexithymia (Grolnick & Ryan, 1989; Joussemet et al., 2008). However, among females, there were no significant effects of PO on alexithymia, which is supported by previous studies with female samples showing no significant relations between PO and alexithymia (De Panfilis et al., 2003; Romeo et al., 2020). Furthermore, no significant indirect effects of PO on the psychological symptoms were found in females. The reason for the differential effects of perceived PO on alexithymia and mental health problems between males and females remains unclear. To some extent, males seem to be more susceptible than females to the parenting style received from fathers during childhood. For example, in a previous study, harsh parenting from fathers affected sons more than daughters, whereas no gender‐related differential effect with mothers' harsh parenting (Chang et al., 2003), which supported our finding that PO had a stronger effect on males' alexithymia and psychological symptoms.

### 4.3 Implications and limitations

The present study explored moderated mediation effects of sex and alexithymia on the relations between perceived parental bonding and depressive/anxiety symptoms in a population‐based birth cohort with relatively large sample size. The findings that alexithymia may function as a mediator accounting for the mental health impacts of parental bonding have implications for understanding the potential mechanism underlying the mental health problems related to childhood parenting experiences. Moreover, compared to females, males unexpectedly appeared to experience more psychological distress related to DysPB through the development of alexithymia, highlighting the significance of paternal bonding in the parenting practice of caregivers. By conducting a moderated mediation model, our study suggests a reasonable explanation regarding how parental bonding experienced during childhood impacts mental health and improves the limited understanding of sex‐specific parental factors for alexithymia and mental health problems, which suggests implications for identifying vulnerable individuals as well as tailoring preventive and psychotherapeutic interventions.

Some limitations in the present study should be acknowledged. First, the self‐report measures may be limited in their validity. Since individuals with alexithymia have difficulty identifying emotions and reflecting on their feelings, future research would benefit from collecting observer‐rated alexithymia data as reference material (Haviland et al., 2000). Additionally, bias might exist due to the retrospective assessment of parental bonding with PBI at a time point different from the measures for alexithymia and the psychological symptoms. Nevertheless, it has been suggested that the PBI is not impacted by the passage of time and mood fluctuations with long‐term stability over 20 years (Murphy et al., 2010). Second, the majority of our study sample is females. Notwithstanding, the larger female sample with higher levels of psychological symptoms even showed a weaker indirect effect of parental bonding when compared with the smaller male sample, so we speculated that the composition of the sample in our study would not bias the outcome of the sex differences.

In addition to the aforementioned limitations, it is important to note that causal inferences cannot be made when interpreting the current findings. Furthermore, complex effects of factors such as genetic influences may be involved in alexithymia and psychological symptoms (Ham et al., 2005; Heiberg & Heiberg, 1977; Jørgensen et al., 2007). Establishing the causality and directionality of the associations is limited by the use of the cross‐sectional study design. In the present study, the lack of repeated measurements and temporal precedence between alexithymia and depressive/anxiety symptoms makes it impossible to verify the effect of alexithymia on the development process of mental health outcomes over time. Although the mediation analysis suggests mechanistic interpretation, there is controversy about whether it is reasonable to determine actual mediation effects using cross‐sectional data (Hayes & Rockwood, 2017; Maxwell & Cole, 2007; Nguyen et al., 2020). Personality–psychopathology theories, although with limited evidence, have posited specific personality traits as predispositions to mental health problems (Gramstad et al., 2013; Jorm et al., 2000; Kotov et al., 2007; Kotov et al., 2010). Alexithymia has been found to be a highly stable personality feature even over 11 years in adult general populations (Hiirola et al., 2017; Tolmunen et al., 2011). Hence, considering the theoretical grounding, we believe that the model‐based findings in this study provide insights into the potential sex‐specific role of alexithymia in the psychological impacts of perceived parental bonding during childhood. However, future research with sophisticated longitudinal designs is strongly recommended for studying the nature of the mediation that may reveal a causal process over time.

## 5 CONCLUSION

The present study indicated that alexithymia may function as a potential mediator for the associations between perceived DysPB during childhood and depressive/anxiety symptoms, which highlights the importance of alexithymia in the parenting‐related mental health impacts. Furthermore, sex differences were confirmed by a moderating role of sex in the relations between DysPB and alexithymia, which, in turn, moderated the alexithymia‐mediated mental health effects of DysPB, with a stronger effect among males. The findings suggest the significance of perceived dysfunctional, especially overprotective paternal bonding during childhood as a noteworthy risk factor for the development of alexithymia and mental health problems in male populations. Longitudinal research that seeks to clarify sex‐specific mediation of alexithymia in a causal process is clearly warranted.

## CONFLICTS OF INTEREST

The authors declare no conflicts of interest.

### PEER REVIEW

The peer review history for this article is available at https://publons.com/publon/10.1002/jclp.23372

## Data Availability

The data are not publicly available due to the federal legal restrictions concerning the data that contains information that could compromise the privacy of research participants. Requests to access the datasets should be directed to the Principal investigator of the FinnBrain Birth Cohort Study (hasse.karlsson@utu.fi).
